# Poisoning by *Baccharis coridifolia* in Early-Weaned Beef Calves: Pathological Study and New Macrocyclic Trichothecene Identification

**DOI:** 10.3390/toxins15120681

**Published:** 2023-12-01

**Authors:** Mizael Machado, Rafael Martínez, Sol Andres, Mark W. Sumarah, Justin B. Renaud, Aníbal G. Armién, Claudio S. L. Barros, Franklin Riet-Correa, Alejo Menchaca, Carlos O. Schild

**Affiliations:** 1 Plataforma de Investigación en Salud Animal (PSA), Instituto Nacional de Investigación Agropecuaria (INIA), Estación Experimental Tacuarembó, Tacuarembó 45000, Uruguay; amenchaca@inia.org.uy (A.M.); schild.co@gmail.com (C.O.S.); 2 Independent Researcher, Tacuarembó 45000, Uruguay; martinezsaralegui@adinet.com.uy; 3 CENUR Noreste, Universidad de la República, Tacuarembó 45000, Uruguay; solandres16@gmail.com; 4 London Research and Development Centre, Agriculture & Agri-Food Canada, London, ON N5V 4T3, Canada; mark.sumarah@agr.gc.ca (M.W.S.); justin.renaud@agr.gc.ca (J.B.R.); 5 California Animal Health & Food Safety Laboratory System (CAHFS), School of Veterinary Medicine, University of California, Davis, CA 95616, USA; agarmien@ucdavis.edu; 6 Faculdade de Medicina Veterinária e Zootecnia (FMZV), Universidade Federal de Mato Grosso do Sul (UFMS), Campo Grande 79070-900, Mato Grosso do Sul, Brazil; claudiobarros1945@gmail.com; 7 Programa de Pós-Graduação em Ciência Animal nos Trópicos, Universidade Federal da Bahia (UFBA), Salvador 49170-110, Bahia, Brazil; franklinrietcorrea@gmail.com; 8 California Animal Health and Food Safety Laboratory System–San Bernardino Branch, University of California, San Bernardino, CA 92408, USA

**Keywords:** Baccharis, *mio-mio*, toxicity, poisonous plants, ruminants, Uruguay

## Abstract

This study investigated two outbreaks of spontaneous poisoning by *Baccharis coridifolia* (Asteraceae) in early-weaned beef calves in Tacuarembó, Uruguay. A total of 34 affected calves showed signs of salivation, anorexia, apathy, marked dehydration, and diarrhea. Deaths occurred 36–72 h after consumption and mortality varied from 37.5% to 43.3% for outbreak 1 and outbreak 2, respectively. The main pathological findings include diffuse severe necrosis of the prestomachs and lymphoid tissues. Ultrastructurally, epithelial cells of the rumen showed swelling, lysis of the organelles, degradation of intercellular attachments, and degradation of the nuclear chromatin. Using LC-MS with diagnostic fragmentation filtering, 56 macrocyclic trichothecenes including glycosyl and malonyl conjugates were identified. The total concentration of macrocyclic trichothecenes, including conjugates, was estimated to be 1.2 ± 0.1 mg/g plant material. This is the first report of these malonyl–glucose conjugates from *Baccharis coridifolia*.

## 1. Introduction

*Baccharis coridifolia* (Asteraceae), usually known as *mio-mio* (in Spanish), is an important highly toxic plant for herbivorous animals that grows in native grasslands in Argentina, Brazil, Uruguay, and Paraguay, causing high mortality within a few hours after consumption [1,2,3]. Macrocyclic trichothecenes constitute a large group of mycotoxins associated with *Baccharis artemisioides*, *Baccharis coridifolia*, and *Baccharis megapotamica* (Table 1). The first report of the detection of macrocyclic trichothecenes in *Baccharis coridifolia* was made by Busam and Habermel, in 1982. Subsequent isolation and characterization showed that roridins, verrucains, and myophytocenes are the most relevant toxins [4,5]. These compounds are highly cytotoxic, causing damage to the cellular membrane, mitochondrial dysfunction, inhibition of protein synthesis, and apoptosis. Other biological activities include antifungal, antibiotic, antimalarial, antiviral, and antitumor activities [6,7,8,9,10,11]. 

*Baccharis coridifolia* can induce conditioned food aversion when ingested in non-toxic amounts by animals. Intoxication usually occurs when animals raised in areas where the plant does not exist are moved to areas where the plant grows and, in some instances, when prolonged fasting occurs prior to exposure. Because of this, cattle from locations where the plant is less abundant are more susceptible to poisoning [2]. Affected animals show signs of salivation, abdominal pain, dehydration, and diarrhea, followed by death. *Baccharis coridifolia* poisoning is characterized pathologically by severe necrotic gastroenteritis and lymphoid necrosis [1,2,15]. However, the pathogenic mechanism of the disease has not been elucidated [14,16]. The determination of macrocyclic trichothecenes and the ultrastructural lesions in spontaneous outbreaks of poisoning have not yet been reported. This study aimed to describe the toxin chemistry of *Baccharis coridifolia* and the epidemiological and clinicopathological aspects of calves naturally exposed to this plant. 

## 2. Results

### 2.1. Case Studies 

**Figure 1:**
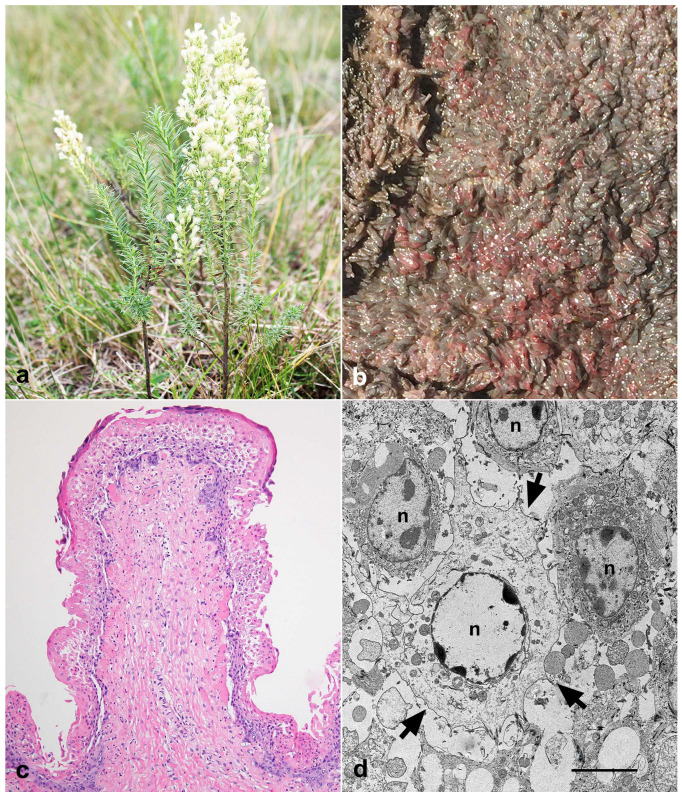
*Baccharis coridifolia* poisoning in early-weaned beef calves. (**a**) *Baccharis coridifolia* plant. (**b**) Red discoloration of the ruminal papillae. (**c**) Necrotic rumenitis, acute, diffuse, accentuated, with areas of detachment of the epithelial layers associated with neutrophil infiltration, H&E, 400×. (**d**) Epithelial cells of the rumen with marked swelling, nuclear degradation, and cellular detachment (arrows). n: nucleus. Transmission Electron Microscopy, bar = 1 μm.

Outbreak 1 occurred in a herd of 160 male early-weaned calves aged 3 months. The animals were Aberdeen Angus, Hereford, or crossbred from the Department of Treinta y Tres (78/160) and San José (82/160). The animals had been on the property for one month and were raised in a paddock cultivated with *Trifolium pratense* (red clover) and *Chichorium intybus* (chicory). They were enclosed overnight in the corral, and in the morning, before entering the paddock, they received commercial feed. Accidentally, a corral gate was left open during the night, and the calves had access to a paddock adjacent to the natural field corral invaded by *Baccharis coridifolia* and shortage pasture (Figure 1a). In the morning, four dead animals were found. Within 36 h, another 14 animals showed clinical signs, and all died. All 18 dead calves came from the Department of Treinta y Tres. 

Outbreak 2 occurred in a herd of 60 male 3-month-old Aberdeen Angus, Hereford, and crossbred calves coming from the Department of Rocha. The animals were placed in the paddock where outbreak 1 occurred to allow them to ingest small non-toxic amounts of this plant through grazing control and develop conditioned food aversion. However, 4–6 h later, they showed clinical signs and 16 animals died within 48–72 h.

### 2.2. Botanical Identification 

The plant was identified as *Baccharis coridifolia* DC. and deposited with the registration number “FRC/04 2022-Uy” in the herbarium Ing. Ag Bernardo Rosengurtt, Botanic Laboratory, Agronomy Faculty of the Universidad de la República in Montevideo, Uruguay.

### 2.3. Clinical and Pathological Data

The observed clinical signs included salivation, anorexia, apathy, enophthalmos, delayed skin turgor, dyspnea, bloat, diarrhea, sternal and lateral recumbency, and prostration. 

Two calves were necropsied (outbreak 1). Macroscopically, petechiae and multifocal ecchymoses were observed in both animals on the serosa of the rumen, indicating moderate edema of the wall of the rumen pillars and adjacent areas. The rumen was full of abundant liquid content. The mucosa of the rumen (Figure 1b), reticulum, and omasum showed focally extensive reddened areas, sometimes with erosions and hemorrhages. The gastric and mesenteric lymph nodes were enlarged, and the cut surface showed multifocally to focally extensive areas of hemorrhage affecting the cortical and medullary areas. In calf 1, the mucosa of the abomasum and the initial portion of the duodenum were diffusely red, with multifocally to focally extensive areas of erosion, sometimes with mucosal detachment. The liver was enlarged and slightly orange. On the capsular surface of the kidneys, there were randomly distributed red punctate areas. In the heart, there were petechiae and ecchymoses distributed over the epicardial surface. Multifocal areas of hemorrhages were observed at the level of the thalamus, the periventricular regions of the rostral and caudal colliculi, and at the pons (calf 1). 

The main histological lesions included necrohemorrhagic, acute, and extensive rumenitis associated with neutrophilic infiltration, edema, and, although more rarely, capillary thrombosis of the submucosa (Figure 1c) and marked lymphatic necrosis characterized by pyknosis and karyorrhexis in germinative centers of the gastric and mesenteric lymph nodes and spleen. Additionally, multifocal hemorrhages in the thalamus and midbrain were observed. Ultrastructurally, swollen epithelial cells of the rumen, degradation of the intercellular junction leading to cell detachment, lysis of the organelles, and degradation of the nuclear chromatin were observed (Figure 1d). 

### 2.4. Phytochemistry Analysis 

**Figure 2:**
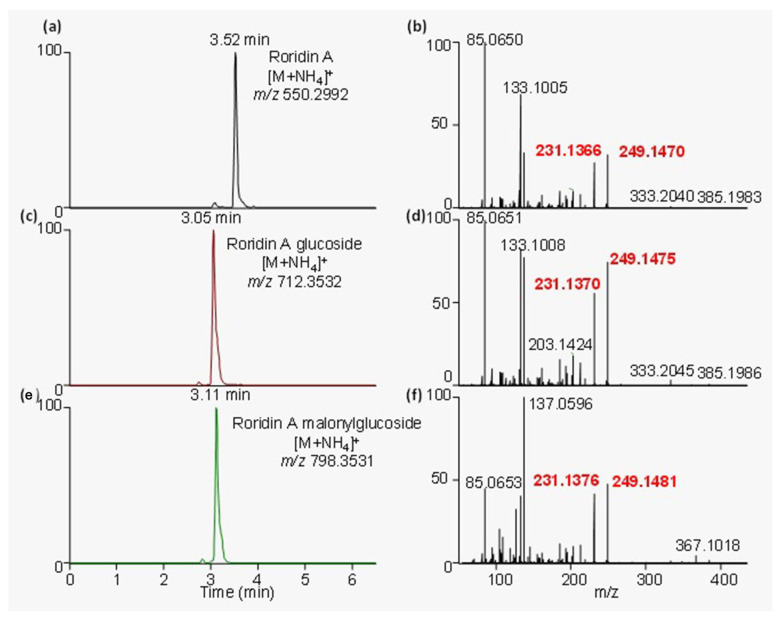
Extracted ion chromatograms and their corresponding MS/MS spectra for (**a**,**b**) roridin A, (**c**,**d**) roridin A–glucoside, and (**e**,**f**) roridin A–malonylglucoside. All these compounds and other macrocyclic trichothecenes share some common fragment ions, specifically at *m*/*z* 231.1376 and 249.1481, which are shown in red text.

Plants samples from these outbreaks were analyzed using a data-dependent acquisition (DDA) method, which collected MS/MS spectra on the most intense ions detected throughout an LC-HRMS run. Fragment ions that were common to all major trichothecenes occurred at *m*/*z* 231.1366 and 249.1470 (Figure 2), in accordance with previous reports [7].

These diagnostic fragment ions, which indicated the presence of a macrocyclic trichothecene, were used within the diagnostic fragmentation filtering (DFF) module of the MZmine 2 software analysis platform to putatively identify all trichothecenes present. Applying DFF to this dataset revealed the presence of 56 potential macrocyclic trichothecenes, including related conjugates. The peak areas of these compounds were determined in each sample by monitoring the [M+H]+ in full MS mode (Table S1). Many of the analytes previously reported in species of *Baccharis* are isobaric. For example, roridin E, miophytocen A, and miophytocen B have the molecular formula C_29_H_38_O_8_. Unambiguous identification within these samples would require analytical standards, which are not commercially available. Within these samples, isobaric compounds are included together as putative. 

### 2.5. Quantification

**Figure 3:**
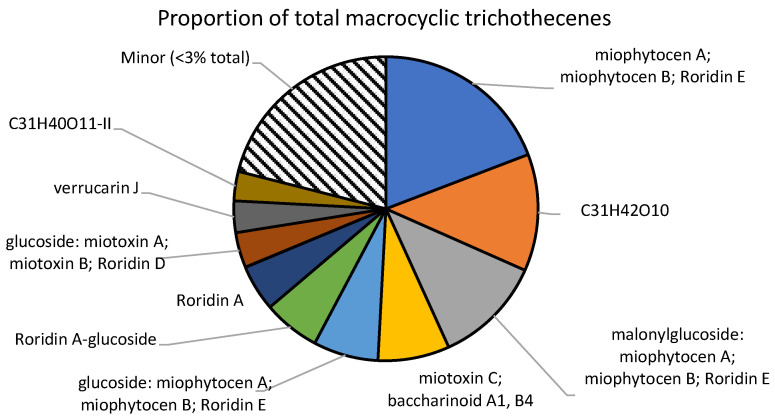
Major macrocyclic trichothecenes detected. All analytes with concentrations below 3% of the total were summed together as “minor”.

**Figure 4:**
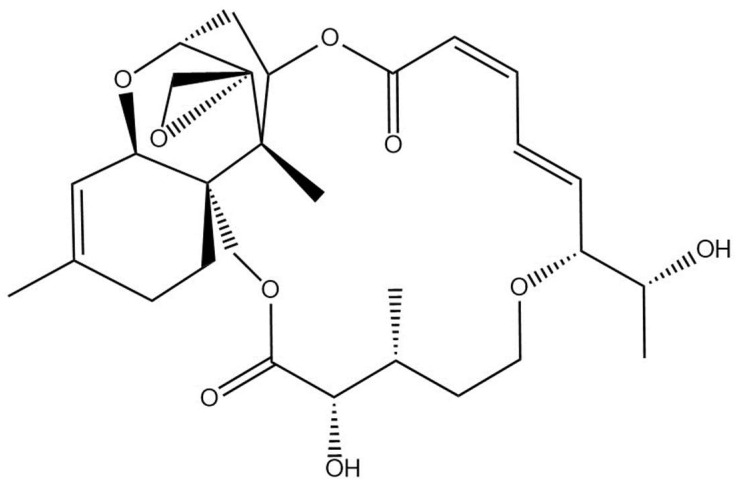
Structure of the roridin A detected in *Baccharis coridifolia*.

As there are no commercially available standards for the numerous detected analytes, the values are reported as “roridin A equivalents”, following the literature procedure [13]. Therefore, except for roridin A, these concentrations represent approximations of the true concentration present. Glycosylated and malonylglycosylated compounds may have starkly different ionization efficiencies, and therefore, the concentrations reported here may be far from their true concentrations (Figure 3). Apo compounds, which do not contain a glucoside or a malonylglucoside, are likely to ionize in a similar manner to roridin A, and the reported concentrations may be close to the true value (Figure 4). The total amount reported within these samples was 1.2 ± 0.1 mg/g plant material, which agrees with literature values [14].

Although 56 distinct macrocyclic trichothecenes were detected in the material, 10 of the analytes made up over 75% of the total amount. The most abundant compound detected was a molecule with the formula C_29_H_38_O_8_, which may correspond to roridin E, miophytocen A, or miophytocen B. An unknown molecule with the formula C_31_H_42_O_10_ was the second most abundant trichothecene, with 149 µg/g plant material. YM-475Z4, a macrocyclic trichothecene with this formula, was previously reported as a fungal metabolite [17]. However, due to a lack of the necessary analytical standards, there is no concrete evidence that the compound detected here was YM-475Z4.

Many of the detected compounds were found to contain a glucose moiety. In addition, some of the molecules were also conjugated to a malonyl–glucose. Although the unconjugated trichothecenes represented 64% of the total amount, glucosides accounted for a significant portion at 21%. Similarly, the malonylglucosides also represented 15% of the total amount. No xylose derivatives that were previously reported in another *Baccharis* species were found within these samples.

## 3. Discussion

Since the 17th century, the toxicity of *Baccharis coridifolia* in southern South America has been well understood from knowledge culturally passed down through generations and livestock owners. However, it is still a limiting factor in ruminant production today. Outbreaks with high mortality often occur when naïve animals are introduced into areas with *Baccharis coridifolia* [1,2]. Cattle belonging to specific areas, such as the departments of Treinta y Tres, Rocha, and Cerro Largo, are less likely to be exposed to *Baccharis coridifolia* and consequently become poisoned when taken to areas where the plant is endemic [2]. In the present cases, the affected animals came from Treinta Tres y Rocha departments.

Affected calves showed salivation, bloat, dyspnea, diarrhea, necrotic gastroenteritis, and lymphoid necrosis. These are hallmarks of *Baccharis* poisoning in ruminants [1,2]. The main ultrastructural lesions observed in the rumen of the affected animals included marked cellular swelling, mitochondrial damage, and nuclear degeneration. These results are similar to previously reported macrocyclic trichothecene toxicity cellular effects [7,8,9,10,18].

There has been an increase in the number of reported macrocyclic trichothecenes in recent decades in *Baccharis* species [6]. In the present study, a total of 56 macrocyclic trichothecenes were detected using LC-MS with diagnostic fragmentation filtering. Many of the trichothecenes detected were conjugated with a hexose sugar. Unlike the previous analysis on *Baccharis megapotamica* [14], no xylose conjugates of the macrocyclic trichothecenes were detected. Interestingly, malonyl–glucose conjugates of several trichothecenes were also detected. The potential application of these new macrocyclic trichothecenes should be investigated. 

We reinforce that cattle are highly susceptible to *Baccharis coridifolia* poisoning unless they have been previously exposed, resulting in conditioned aversion. Introduction of naïve animals into pastures with this plant may result in heavy economic losses. The conditions under which intoxication occurs are particular to *Baccharis* spp. and currently there is not an effective treatment [1]. Controlled grazing, as observed in one of the outbreaks reported here, presents variable results, and the animals can become intoxicated during these practices [1]. Conditioned aversion could be induced by administering a sublethal dose orally. Although this strategy is laborious to use on many animals, it can be applied by owners to avoid high mortalities. Interestingly, to avoid poisoning in animals to be transported, there are records of livestock owners planting *Baccharis coridifolia* in areas considered free of the plant in Uruguay (Mizael Machado, personal communication).

## 4. Materials and Methods

### 4.1. Case History and Clinical and Pathological Investigation

The outbreaks occurred on a farm belonging to the Department of Tacuarembó, Northern Uruguay (32°0′28.098″ S, 55°13′52.709″ W), expecting to receive beef cattle of all ages from different locations in Uruguay. Most of the animals were received for identification and sanitary handling and later transported to other farms. Clinical history was obtained from the livestock owners and veterinarians during visits to the farms to determine the cause of the outbreaks (outbreaks 1 and 2) in early-weaned beef calves. All calves had a regular body condition and were transported by truck for several hours before poisoning. The animals were clinically evaluated during the visits, and two calves (calves 1 and 2—outbreak 1) were necropsied. Organs of the thoracic and abdominal cavities and brain were obtained, fixed in buffered 10% formalin, routinary processed, and stained using the hematoxylin and eosin technique. The *Baccharis coridifolia* samples were collected randomly in the paddock for chemical analysis.

### 4.2. Transmission Electron Microscopy 

Rumen-fixed samples of two calves (outbreak 1) were subjected to Transmission Electron Microscopy examination. Briefly, samples were dehydrated in a 25–100% ethyl alcohol gradient series for 2 h using an automated tissue processor (Leica Microsystems). Tissue was then infiltrated with EMbed 812 resins, embedded, and incubated at 58˚C for 24 h for polymerization (all reagents were from Electron Microscopy Sciences). To select areas of interest, samples were trimmed, sectioned to be 1 micron thin, and stained with toluidine blue. From selected areas, thin sections (70–90 nm) were obtained and contrasted with 5% uranyl acetate and Sato’s lead citrate. To improve contrast, a second grid was used and only contrasted with 5% uranyl acetate. All samples were visualized using a JEOL 1400Plus transmission electron microscope (JEOL LTD, Tokyo, Japan). Images were obtained and analyzed using a OneView Camera Model 1095 with the Gatan Microscope Suite 3.0 (Gatan Inc., Pleasanton, CA, USA).

### 4.3. Phytochemistry Analysis 

Samples were analyzed as triplet sub-samples. A total of 200 mg of material was placed into a microcentrifuge tube, and 1 mL of extraction solvent, comprising water/acetonitrile/acetic acid (20:78:2), was added to the microcentrifuge tubes, which were then vortexed for 15 s. The tubes were then placed in a sonicating water bath for 15 min and held at 30 °C. The samples were vortexed again and placed in a Thermomixer at 1400 rpm and 30 °C for 30 min. The tubes were then centrifuged at 8000× for 8 min at 4 °C. A total of 160 µL of supernatant was removed and diluted with 40 µL of water and placed at 4 °C for 30 min. The liquid was then centrifuged as before, and the supernatant was placed into a 250 µL polypropylene HPLC vial for LC-MS analysis.

High-resolution mass spectrometry (HRMS) data were obtained using a Thermo Q-Exactive Quadrupole Orbitrap Mass Spectrometer (Mississauga, ON, Canada) coupled to an Agilent 1290 HPLC (Mississauga, ON, Canada). For chromatographic separation, a Zorbax Eclipse Plus RRHD C18 column (2.1 × 50 mm, 1.8 µm; Agilent, Mississauga, ON, Canada) was maintained at 35 °C using mobile phases comprising water with 0.1% formic acid (A) and acetonitrile with 0.1% formic acid (B) (Optima grade, Fisher Scientific, Fair Lawn, NJ, USA). Mobile phase B was held at 0% for 30 s and increased to 100% over three and a half minutes. B was held at 100% for one and a half minutes before returning to 0% in 30 s. Injections were made at a volume of 5 µL, and a flow rate of 0.3 mL/min was used. The following conditions were used for positive HESI MS: capillary voltage, 3.9 kV; capillary temperature, 400 °C; sheath gas, 17.00 units; auxiliary gas, 8.00 units; probe heater temperature, 450 °C; S-Lens RF level, 45.00. The samples were analyzed using a data-dependent acquisition approach, which consisted of a full ms scan acquired for a range *m*/*z* 175–1000 at 35,000 resolution, AGC target, 1 × 106, and maximum injection time (IT), 128 ms. The 5 most intense ions were sequentially selected for MS/MS at a resolution of 17,500, AGC target, 5 × 106, maximum injection time (IT), 64 ms isolation window of 1.2 *m*/*z*, and normalized collision energy (NCE) of 30. 

Samples were screened for the presence of both known and unknown trichothecenes using a diagnostic fragmentation filtering (DFF) approach, which identified all MS/MS spectra that contained both the m/z 231.1366 and 249.1470 product ions [19,20]. As we lacked authentic standards for every putatively identified macrocyclic trichothecene, quantification was performed using a commercial roridin A standard. Final reported concentrations are thus reported as roridin A equivalents following the procedure of Oliveira-Filho et al., 2012 [14]. 

## Figures and Tables

**Table 1:** Data from *Baccharis* species, chemistry, and features associated with poisoning in livestock.

Plants	Regions	Affected Species	Methodology	Mycotoxin	Pathology	Main References
*Baccharis* *artemisioides*	North and west of Buenos Aires and southeast of Cordoba in Argentina	Cattle, sheep, and horses	Thin-layer chromatography	Roridins and verrucarins	Necrotic gastroenteritis and lymphoid necrosis	[12]
	Limited distribution (drained soils)					
*Baccharis* *coridifolia*	Argentina, Southeast and Southern Brazil, Uruguay, and Paraguay	Cattle, sheep, and horses	Two-dimensional Fourier transform NMR Thin-layer chromatography	Roridins, verrucarins, and miophytocenes	Necrotic gastroenteritis and lymphoid necrosis	[4,5,13]
	Wide distribution (drained soils)					
*Baccharis* *megapotamica*	Southern Brazil	Cattle, goats, and buffaloes	UHPLC with high-resolution time of flight mass spectrometry and tandem mass spectrometry	Baccharin, Baccharinoid, and roridins	Necrotic gastroenteritis and lymphoid necrosis	[13,14]
	Limited distribution (moist soils)					

## Data Availability

The data presented in this study are available in this article.

## Supplementary Materials

The following supporting information can be downloaded at: https://www.mdpi.com/article/10.3390/toxins15120681/s1. Table S1: detected analytes and their concentration in “roridin A equivalents”.
