# Supplementary material for: Poisoning by Baccharis coridifolia in Early-Weaned Beef Calves: Pathological Study and New Macrocyclic Trichothecene Identification
Source: Toxins (Basel). 2023 Dec 1;15(12):681. doi: 10.3390/toxins15120681 (PMC10747622; doi:10.3390/toxins15120681)
Supplement: Supplementary file 1 [file toxins-15-00681-s001.zip › toxins-2672726-supplementary.pdf]

toxins

MDPI

Table S1. – Detected analytes and their concentration in 'roridin A equivalents'. All compounds contained fragment ions at m/z 231.1366 and 249.1470 that are common to all major trichothecenes. (SD = standard deviation).

		Apo			
#	***Putative compounds***	***m/z***	***RT (min)***	***ug/g***	*SD +/-*
1	verrucarin J	502.2435	4	40	*4*
2	verrucarin A	520.2541	3.37	0.26	*0.09*
3	*iso*-verrucarin A	520.25411	3.55	0.23	*0.2*
4	roridin H; verrucarin H	530.2748	4.05	<LOD	-
5	roridin H; verrucarin H	530.2748	3.8	<LOD	-
6	miophytocen A; miophytocen B; roridin E	532.2905	3.91	230	*25*
7	miophytocen A; miophytocen B; roridin E	532.2905	3.74	1.9	*0.2*
8	miotoxin A; miotoxin B; roridin D	548.2854	3.46	20	*2*
9	miotoxin A; miotoxin B; roridin D	548.2854	3.64	11	*1*
10	miotoxin A; miotoxin B; roridin D	548.2854	3.7	19	*2*
11	miotoxin D; miotoxin iso-D	550.3012	3.07	0.85	*0.1*
12	roridin A	550.3011	3.51	59	*6*
13	baccharinoid B27	562.2647	3.72	<LOD	-
14	miotoxin E; baccharinoid B27, B10, B12, B17, B21, B9, B13, B14, B16	564.2803	3.38	16	*1*
15	miotoxin F; baccharinoid B1, B2, B3, B7, B20, B23, B24	566.2959	3.27	5.1	*0.4*
16	baccharin; baccharinoid B5, baccharinol, isobaccharinol, isobaccharin	580.2752	3.07	<LOD	-
17	miotoxin C; baccharinoid A1, B4	608.3065	3.57	91	*3*
18	miotoxin C; baccharinoid A1, B4	608.3065	3.43	1.0	*0.3*
		**glucosides**			
	Putative compounds	***m/z***	***RT (min)***	***ug/g***	**+/-**
19	miophytocen A; miophytocen B; roridin E	694.3433	3.2	2.4	*0.4*
20	miophytocen A; miophytocen B; roridin E	694.3433	3.29	82	*9*
21	miophytocen A; miophytocen B; roridin E	694.3433	3.75	<LOD	-
22	miotoxin A; miotoxin B; roridin D	710.3382	3.08	14	*3*
23	miotoxin A; miotoxin B; roridin D	710.3382	3.15	45	*5*
24	roridin A	712.3539	3.05	72	*10*
25	miotoxin D; miotoxin iso-D	712.3539	2.74	0.62	*0.2*
26	miotoxin E; baccharinoid B27, B10, B12, B17, B21, B9, B13, B14, B16	726.3331	3.01	16	*1*
27	miotoxin F; baccharinoid B1, B2, B3, B7, B20, B23, B24	728.3488	2.97	7.7	*1*
		**malonyl glucoside**			
	Putative compounds	***m/z***	***RT (min)***	***ug/g***	**+/-**
28	verrucarin A	768.3073	3.13	1.2	*0.5*
29	roridin H; verrucarin H	778.3281	3.25	<LOD	-
30	miophytocen A; miophytocen B; roridin E	780.3437	3.27	3.7	*0.6*
31	miophytocen A; miophytocen B; roridin E	780.3437	3.36	139	*18*
32	miotoxin A; miotoxin B; roridin D	796.3386	3.14	2.1	*0.4*
33	miotoxin A; miotoxin B; roridin D	796.3386	2.93	<LOD	-
34	miotoxin A; miotoxin B; roridin D	796.3386	3.23	11	*2*
35	roridin A	798.3543	3.11	19	*3*

36	miotoxin D; miotoxin iso-D	798.3543	2.81	<LOD	-
37	miotoxin E; baccharinoid B27, B10, B12, B17, B21, B9, B13, B14, B16	812.3335	3.07	<LOD	-
38	miotoxin F; baccharinoid B1, B2, B3, B7, B20, B23, B24	814.3492	3.02	<LOD	-
		**Unknowns**			
		**apo**			
	Putative compounds	***m/z***	***RT (min)***	***ug/g***	**+/-**
39	Unk-C26H34O7	476.2643	4.37	0.46	*0.2*
40	Unk-C31H38O10-I	588.2803	3.93	6.7	*0.4*
41	Unk-C31H38O10-II	588.2803	4.01	1.6	*0.2*
42	Unk-C31H40O10-I	590.2960	3.8	14	*0.4*
43	Unk-C31H40O10-II	590.2960	3.98	20	*13*
44	Unk-C31H40O10-III	590.2960	4.03	26	*9*
45	Unk-C31H40O10-IV	590.2960	4.21	2.7	*0.2*
46	Unk-C31H40O11-I	606.2909	3.64	2.2	*0.2*
47	Unk-C31H40O11-II	606.2909	3.73	38	*0.4*
48	Unk-C31H40O9-I	574.3011	4.17	1.8	*0.1*
49	Unk-C31H40O9-II	574.3011	4.26	6.6	*0.5*
50	Unk-C31H42O10	592.3116	3.87	149	*7*
51	Unk-C33H44O11	634.3222	4.17	8.2	*0.3*
		**glucoside**			
	Putative compounds	***m/z***	***RT (min)***	***ug/g***	**+/-**
52	Unk-C31H40O10-I	752.3488	3.25	<LOD	-
53	Unk-C31H40O10-II	752.3488	3.35	<LOD	-
54	Unk-C31H40O9-I/II	736.3539	3.53	5.6	*0.8*
55	Unk-C31H42O10	754.3646	3.25	4.5	*0.5*
		**malonyl glucoside**			
	Putative compounds	***m/z***	***RT (min)***	***ug/g***	**+/-**
56	Unk-C31H42O10	840.3648	3.32	0.72	*0.1*
**Total (mg/g)**				1197	*136*
